# Concurrent Taxol-Based Doublet Chemotherapy as an Alternative for Intracavitary Brachytherapy Boost in Locally Advanced Carcinoma Cervix- Retrospective Analysis From a Tertiary Care Center

**DOI:** 10.7759/cureus.16313

**Published:** 2021-07-11

**Authors:** Christopher John, Balasubramanian Venkitaraman, Hemavathi Masilamani, Satish S Kondaveeti

**Affiliations:** 1 Radiation Oncology, Sri Ramachandra Institute of Higher Education and Research, Chennai, IND; 2 Surgical Oncology, Sri Ramachandra Institute of Higher Education and Research, Chennai, IND

**Keywords:** carcinoma cervix, ebrt boost, taxol/carboplatin, doublet, locally advanced

## Abstract

Objective

In light of the dogma that brachytherapy is irreplaceable for the successful treatment of cervical cancer, and the limited availability of brachytherapy facilities in developing countries, we sought to evaluate the toxicity and efficacy of taxol- and platinum-based doublet chemotherapy delivered concurrently with external beam radiotherapy (EBRT) in locally advanced cervical cancer as an alternative to brachytherapy, which is mandated as the standard of care according to current guidelines.

Methods

The records from our institution were reviewed to identify patients who underwent chemoradiation with two doses of tri-weekly docetaxel (80mg/m2) and carboplatin (AUC 5) concurrent with EBRT between January 2017 and 2019 for locally advanced cervical carcinoma. Here, 48 cases were analysed, with a median follow-up period of two years.

Results

The two groups were homogenously matched, and the patients who received EBRT boost and brachytherapy boost achieved complete pathological response rates of 68% and 83%, respectively (p=0.243). The odds ratio was 0.45 (95% confidence interval, 0.09-2.08), indicative of non-significance and non-inferiority based on the analysis using the chi-squared test (with Pearson’s correlation) and Student's t-test. The disease-free survival durations calculated using Kaplan-Meier estimates were 22 and 24 months, two-year disease-free survival rates were 83% and 91.3%, and two-year overall survival (OS) were 85.6% and 94% for the EBRT boost and brachytherapy boost groups, respectively (p=0.657).

Conclusion

In this retrospective analysis, we concluded that EBRT boost was non-inferior to brachytherapy boost and could be considered as a reasonable alternative in locally advanced cervical cancer when used concurrently with more dose-intense chemotherapy.

## Introduction

Cervical cancer is the third commonest cause of cancer-related mortality among Indian women. India accounts for approximately 25% of the global burden of cervical cancer, and 17% of cervical cancer-related deaths occur among women aged 30-69 years [1]. Statistical data suggest that approximately 527,624 new cervical cancer cases are included in the Globocan database annually worldwide. India alone contributes approximately 122,844 cervical cancer cases annually [2].

Surgery is the preferred modality for stages IA-IIA1 of cervical cancer, while locally advanced cases (stages IIA2-IIIC) are treated with concurrent chemoradiation, which usually includes external beam radiation with concurrent chemotherapy followed by intracavitary brachytherapy. Concurrent platinum-based chemotherapy has been combined with radiotherapy based on the evidence from two meta-analyses that confirmed the survival benefits of concomitant administration of platinum-based chemotherapy and radiation [3-4].

However, in reality, do all patients with locally advanced cervical cancer (International Federation of Gynecology and Obstetrics [FIGO] stages IIB-IIIC2) receive brachytherapy? The answer is no. In our clinical practice, we often encounter patients with locally advanced cervical cancer who have received concurrent chemoradiation with weekly cisplatin and are not found suitable for intracavitary brachytherapy after receiving 50 Gy of external beam radiotherapy (EBRT) due to residual gross central and/or parametrial disease. These patients typically continue EBRT with a reduction in radiation field size to minimize bowel toxicity. Other factors that contribute to ineligibility for brachytherapy are physical considerations that prevent applicator placement, such as decreased vaginal accommodation, cervical canal, and retroversion of the uterus, which may be congenital or age- or disease-related.

In addition, an important emerging factor is the lack of availability of brachytherapy modalities in many radiation centers in developing nations. According to data from 2019, India had a total of 545 teletherapy units and 256 brachytherapy units (250 high dose rate and 6 low dose rate units) [5]. Brachytherapy is an integral part of cervical cancer radiation and remains an important reason for the high local control rates seen traditionally with chemoradiation. However, to date, not all radiation centers in India provide this important modality; hence, patients undergoing external beam radiation therapy at such centers need to be referred elsewhere to undergo brachytherapy. Unfortunately, due to socioeconomic reasons, some of these patients do not receive brachytherapy, resulting in incomplete therapy and local failure. Although the lack of availability of brachytherapy cannot be justified as a valid reason for denying brachytherapy, it is a miscellaneous factor that can affect the outcome of therapy.

In light of the dogma that brachytherapy is irreplaceable for successful treatment of cervical cancer with radiotherapy, some studies have suggested using stereotactic body radiation therapy (SBRT) boost in patients who could not receive brachytherapy for the above-mentioned reasons [6]. Here again, one must remember that not all centers in India have SBRT facilities. Therefore, how do we treat these patients with bulky locally advanced cervical cancer without brachytherapy or SBRT?

We propose the use of dose-intense chemotherapy with EBRT as a clinically effective alternative to brachytherapy. Carboplatin is an alkylating agent with comparable efficacy to cisplatin and provides a favorable toxicity profile with regard to nephrotoxicity and less emetogenic properties. Paclitaxel is a naturally occurring taxane that potentiates radiation-induced damage by binding to beta-tubulin and induces cell death in the cell cycle phase of G2/M [7]. In locally advanced cervical cancers, combining both taxol and carboplatin with definitive radiotherapy produced a clinical response in 80% of patients, with favorable toxicity (comprising grade III gastrointestinal and hematological toxicity) in the range of 9-50% of patients in the literature reviewed [8]. Although the evidence on the use of taxol/platinum in the concurrent setting is sparse, there is abundant evidence for using doublet chemotherapy [9]. It has been suggested that induction chemotherapy or concurrent doublet taxol-based chemotherapy can increase radiosensitivity and decrease the hypoxic cell fraction. Moreover, it has the ability to treat micrometastatic disease, thereby preventing a significant proportion of distal relapses [10].

Hence, this retrospective study was conducted to evaluate the toxicity and efficacy of taxol and platinum-based doublet chemotherapy delivered concurrently with EBRT in locally advanced cervical cancer. We also performed a subanalysis of the potential role of this chemoradiation protocol in improving local disease control as an alternative to brachytherapy, which is mandated as the standard of care according to current guidelines.

## Materials and methods

Materials and methods

We performed a retrospective analysis of the data of patients who underwent treatment between January 2017 and January 2019 at our institution for locally advanced cervical carcinoma. Our records were reviewed to identify patients who received chemoradiation with two doses of tri-weekly taxol and carboplatin concurrent with EBRT (a dose of 50.4 Gy in 28 fractions with 3D conformal radiation therapy). After obtaining approval from our institutional ethics committee, we reviewed the data of patients with histologically confirmed squamous cell carcinoma or adenocarcinoma of the uterine cervix with stages IIB to IIIC according to the 2018 International Federation of Gynecology and Obstetrics (FIGO) classification and an Eastern Cooperative Oncology Group (ECOG) performance status of less than or equal to 2. Patients were ineligible if they had a history of other malignancies or prior history of radiotherapy/chemotherapy and recurrent cervical lesions.

In this study, the data were analyzed retrospectively from a prospectively collected database of another study that evaluated the need for hysterectomy after definitive radiotherapy. Pre-treatment evaluation included history taking, physical examination, hematological evaluation, serum biochemistry, staging, and metastatic workup with a chest X-ray and computed tomography scan of the abdomen and pelvis.

Treatment protocol

Radiotherapy using 3D conformal EBRT was delivered at a dose of 5040 cGy in 28 fractions to the entire pelvis, followed by an EBRT boost of 1080 cGy in 5 fractions for a total dose of 6120 cGy with a reduced field in patients who were deemed unfit for brachytherapy either due to residual parametrial disease or gross central disease with endocervical component post-EBRT. Patients who were deemed fit for brachytherapy were subjected to intracavitary brachytherapy application with 750 cGy (high dose radiotherapy) × 3 fractions with an inter-fraction interval of seven days. The rectum and bladder doses (d2cc) were restricted to less than 65Gy and 75Gy respectively.

Concurrent chemotherapy was administered once every 21 days with an injection of docetaxel (80 mg/m2) and carboplatin (area under the curve [AUC] = 5). Two cycles of chemotherapy were delivered concurrently with radiotherapy on days 1 and 21 with appropriate antiemetics and granulocyte colony-stimulating factor support. The dose of carboplatin was calculated using the Calvert formula:

Total carboplatin dose (mg) = AUC (target dose level) × (glomerular filtration rate + 25).

Response/toxicity

Toxicity profiles were evaluated using the Common Terminology Criteria for Adverse Events. Tumor response was assessed pathologically.

Statistical analyses

Continuous and categorical data were analyzed using the Student t-test and chi-squared test, respectively. The overall survival (OS) and disease-free survival (DFS) curves were estimated using the Kaplan-Meier method and compared using the log-rank test. The collected data were fed into the software after proper validation, error checks, and analyzed using SPSS version 25 (IBM Corporation, Armonk, NY). For all analyses, p<0.05 were considered statistically significant.

## Results

Patient characteristics

At the time of the analysis, the median follow-up period was two years. Our final data included 48 cases, which were retrospectively analyzed. These patients were divided into two groups based on whether they underwent brachytherapy after EBRT or whether they underwent EBRT boost. 

Table 1 shows the characteristics of the patients in this study and the comparison of patient data between the two groups. The analysis revealed squamous cell carcinoma as the predominant histology, while stage IIIC1 was the dominant disease stage. All patients had an ECOG performance status of 0-1. The clinicopathological characteristics were well-balanced, and there were no major differences in age distributions, stage, histology, and tumor grade. A comparable median overall treatment time of 54-57 days was noted. A total of 25 (52.1%) patients had undergone EBRT boost, and 23 (47.9%) patients had undergone brachytherapy boost. Patients in both groups were comparable in terms of age, histology, and tumor grade. However, the EBRT boost group had more locoregionally advanced disease, with 18 patients (72%) with stage III disease and the maximum tumor dimension compared to 13 patients (56.5%) with stage III disease in the brachytherapy boost group.

**Table 1:** Patient's characteristics Values are presented as number (%) FIGO: International Federation of Gynaecologic Oncology; EBRT: external beam radiotherapy

Characteristics	All patients (n=48)	No of patients (n=25) EBRT boost	No of patients (n=23) brachytherapy boost	p-value
Median age, years	52 (37–67)	52 (37–67)	52 (40–65)	0.939
FIGO stage (2018)				
IIB	17 (35.4%)	7 (28%)	10 (43.5%)	0.721
IIIA	2 (4.2%)	1 (4%)	1 (4.3%)	
IIIB	4 (8.3%)	2 (8%)	2 (8.7%)	
IIIC1	25 (52.1%)	15 (60%)	10 (43.5%)	
Histology				
Squamous cell carcinoma	40 (83.3%)	19 (76%)	21 (91.3%)	0
Adenocarcinoma	5 (10.4%)	4 (16%)	1 (4.3%)	
Mixed histology	3 (6.3%)	2 (8%)	1 (4.3%)	
Tumor grade				
I	-	-	-	0.031
II	42 (87.5%)	22 (88%)	20 (87%)	
III	6 (12.5%)	3 (12%)	3 (13%)	

Chemotherapy tolerance and toxicity

The patients underwent a median of two cycles, which was tolerated by 96% of the study population (Table 2). The patients were given prophylactic growth factor support and appropriate anti-emetic agents. However, four patients had allergic reactions to the first dose of taxane-based chemotherapy, which resulted in a change in the chemotherapy schedule to 5-fluorouracil + cisplatin.

**Table 2:** Chemotherapy tolerance Values are presented as number (%)

Chemotherapy regimens	No. of patients
Taxanes/carboplatin (thrice weekly)	44
5-fluorouracil + cisplatin	4
No of cycles	
1	2 (4%)
2	46 (96%)

The commonest acute toxicity was hematological (Table 3), which was managed with appropriate supportive care and did not result in prolonged treatment interruption. Only nine (36%) patients in the EBRT boost group and six (26%) in the brachytherapy boost group had grade III/IV hematological toxicity. Among them, two patients were restricted to one cycle of chemotherapy due to persistent neutropenia and the other patient had an underlying autoimmune disorder which resulted in poor tolerance of chemotherapy. The commonest gastrointestinal toxicity (Table 4) was grade II (72% vs 52.2% in the EBRT boost and brachytherapy boost groups, respectively).

**Table 3:** Hematological toxicity Values are presented as number (%); EBRT: external beam radiotherapy

Grade of hematological toxicity	EBRT boost	Brachytherapy boost
I	11 (44%)	9 (39.1%)
II	5 (20%)	8 (34.9%)
III	7 (28%)	5 (21.7%)
IV	2 (8%)	1 (4.3%)

**Table 4:** Gastro-intestinal toxicity Values are presented as number (%); EBRT: external beam radiotherapy

Grade of gastrointestinal toxicity	EBRT boost	Brachytherapy boost
I	4 (16%)	9 (39.1%)
II	18 (72%)	12 (52.2%)
III	3 (12%)	2 (8.7%)
IV	- (0%)	- (0%)

Treatment outcomes

Among the patients who underwent EBRT boost, 17 (68%) achieved a complete pathological response, while eight patients (32%) had a partial response with evidence of residual pathological disease. In comparison, among the patients who received brachytherapy boost, 19 (83%) achieved a complete pathological response, while four patients (17%) had a partial response with evidence of residual pathological disease as listed in Table 5. It is noteworthy that the EBRT boost group had a greater number of patients with locoregionally advanced disease with a maximum tumor dimension of 6.5 × 5.2 cm (mean, 5 cm). The odds ratio was 0.45 (95% confidence interval, 0.09-2.08), indicative of non-significance and non-inferiority based on the analysis.

**Table 5:** Distributions of patients’ clinical responses to EBRT boost and brachytherapy boost Values are presented as number (%) EBRT: external beam radiotherapy; CR: complete response; PR: partial response

Overall/Stage-wise responses	EBRT boost (n=25)	Brachytherapy boost (n=23)	p-value
Overall responses			
CR	17 (68%)	19 (82.6%)	0.243
PR	8 (32%)	4 (17.4%)	
Stage-wise response			
IIB	n=7	n=10	
CR	5 (71.4%)	9 (90%)	0.323
PR	2 (28.6%)	1 (10%)	
IIIA–C	n=18	n=13	
CR	11 (61.1%)	10 (76.9%)	0.353
PR	7 (38.9%)	3 (23.1%)	

**Figure 1:**
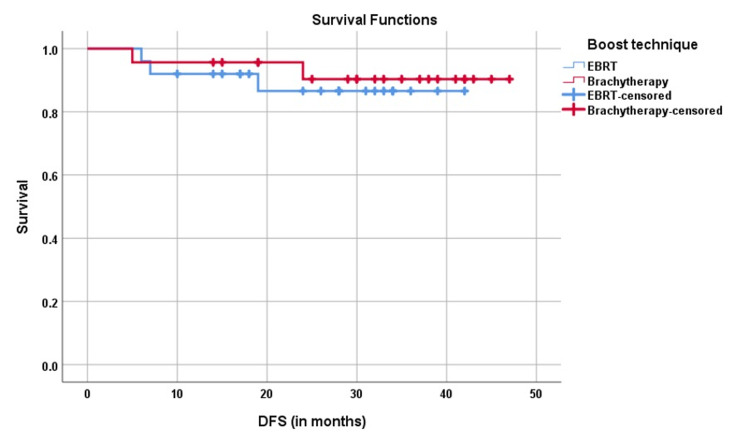
Kaplan-Meier analysis of two-year disease-free survival DFS: disease-free survival; EBRT: external beam radiotherapy

**Figure 2:**
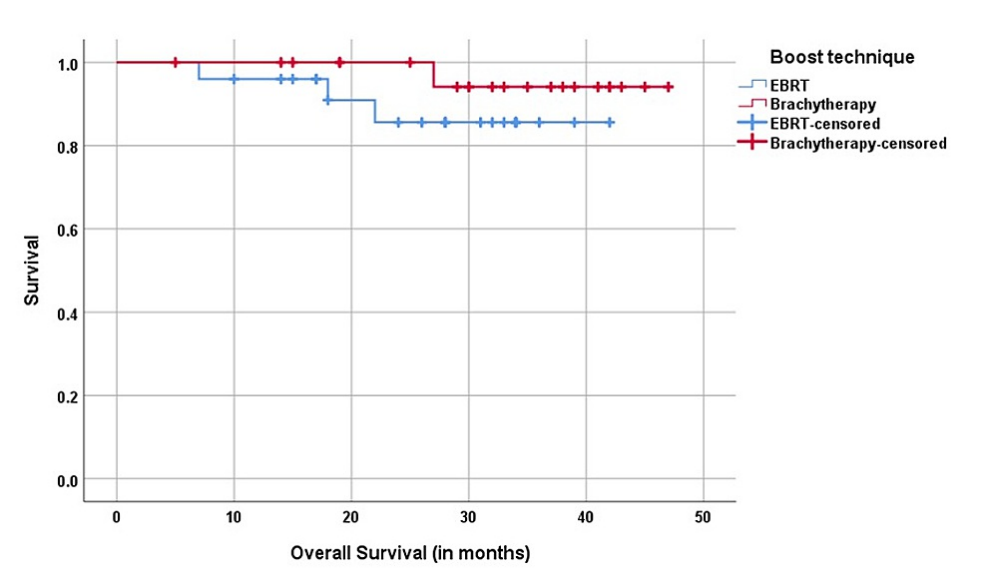
Kaplan-Meier analysis of overall survival OS: overall survival; EBRT: external beam radiotherapy

The median DFS was 22 and 24 months, and the 2-year DFS was 83% and 91.3% and 2-year OS was 85.6% and 94% in the EBRT boost and brachytherapy boost groups, respectively (p=0.657) (Figures 1-2).

## Discussion

The standard of care for locally advanced cervical carcinoma involves administering external beam radiation to the pelvis, including the primary and regional nodes, for a total dose of 45-50 Gy with concurrent cisplatin, followed by a boost dose to the primary tumor using intracavitary brachytherapy for a total dose of 80-85 Gy to point A. The incorporation of chemotherapy concurrent with EBRT along with brachytherapy has played an integral role in improving local control and OS. However, not all patients who undergo initial EBRT are fit to undergo brachytherapy due to anatomical considerations or residual tumor extent. Such patients were subjected to further EBRT up to 60 Gy with a reduced field. Nonetheless, most patients do not attain complete response and end up with salvage surgeries for residual/recurrent local disease.

High-precision radiotherapy techniques to boost the central dose are alternatives to brachytherapy, as the total dose that could be delivered using the 3D conformal or conventional 4-field box technique has always been limited by the tolerance of surrounding organs at risk, including the rectum, bladder, and bowel. The use of techniques such as intensity-modulated radiation therapy [11] and stereotactic radiotherapy (SRT) [6] has enhanced the dose delivered to the residual tumor volume while sparing the normal tissue from receiving high doses. In a study conducted by Marnitz et al. in which SRT boost was used as an alternative to brachytherapy (wherein 6 Gy × 5 fractions were delivered as a boost), the researchers found a favorable toxicity profile with a complete response rate of 81.8% [12-15]. Therefore, these techniques could potentially be a suitable substitute for brachytherapy and would increase the chance of local control and survival while reducing toxicity in patients who could not be candidates for brachytherapy. In a study conducted by Jorcano et al., the use of SRT boost was hypothesized and yielded three-year loco-regional failure-free survival and OS rates of 96% and 95%, respectively [6]. However, access to these high-precision radiotherapy techniques with their limited availability is beyond the reach of most patients and hence may not apply to patients with limited logistical resources in developing countries. 

Against this background, our study attempted to analyze the benefit of taxol/platinum doublet chemotherapy used concurrently with EBRT as a cost-effective alternative to brachytherapy and high-precision radiotherapy, particularly in resource-limited settings. We aimed to analyze whether an increase in chemotherapy intensity when combined with external beam radiation might translate into better local control in this subset of patients who are unfit for or unable to access brachytherapy. The study also compared the stage-wise pathological response rate of this approach with that of the standard of care, which is EBRT followed by intracavitary brachytherapy.

The supporting literature for our concept was drawn from the study conducted by Higgins et al. in 2003, in which the use of concurrent taxol/platinum doublet with radiotherapy was evaluated in locally advanced cervical carcinoma and was suggestive of complete response rates of 91% at three months post-therapy and three-year PFS and OS rates of 70% and 65%, respectively which was comparable to the data achieved in our study [16]. Their study found a favorable toxicity profile, in which grade III/IV rates were unusual due to a low dose weekly regimen with paclitaxel administered for a dose of 40mg/m2. However, in comparison, our study had more grade III rates probably owing to the more dose intense chemotherapy regimen which however did not translate to any major treatment interruptions [16]. Other phase I trials in the literature reviewed for locally advanced cervical carcinoma also substantiated the benefit of doublet chemotherapy with radiotherapy with weekly paclitaxel/carboplatin chemotherapy [17,18]. Rao et al. reported that the maximum tolerated doses were carboplatin AUC 2.5, with paclitaxel 50 mg/m2 weekly with respect to hematological toxicity, wherein grade 3/4 nonhematological toxicities were rare. The two-year DFS and OS rates were 80% and 86%, respectively. The main lacuna of their study is the limited sample size [19]. 

A study conducted by Tripathi et al. in 2019 used an induction chemotherapy doublet with taxol/platinum followed by concurrent chemoradiation, which showed an overall response rate of 96% comparable to our study, with a two-year DFS rate of 91.3% and OS of 94%. The occurrence of grade III hematological toxicities reported was 12.5% of patients in the study group [20]. The novelty of our study lay in that we used the chemotherapy component from the induction arm in the concurrent setting while maintaining a favorable toxicity profile with grade III toxicities limited to 18.75% and grade IV toxicities seen in 6.25% of subjects. 

Our study had some limitations, as it was retrospective, and the disease burden with respect to the disease stage was not evenly matched between the two arms. However, we have to admit that the EBRT boost group had more patients with advanced disease and still achieved a pathological complete response in >65% of patients without brachytherapy. This can be explained by considering that the increased intensity of chemotherapy, when administered concurrently with EBRT, increases the biologically effective dose, which can compensate to a certain extent for the dose intensity provided by brachytherapy. However, this hypothesis is vague and we need a properly designed prospective randomized control trial with suitable statistical strength to substantiate the preliminary data obtained in this study. 

## Conclusions

Our study may be an eye-opener that highlights that a more intense chemotherapy regime when employed concurrently with EBRT, followed by EBRT boost in locally advanced cervical cancer, could be a practical alternative for patients who may not have access to brachytherapy facilities.
